# HumCFS: a database of fragile sites in human chromosomes

**DOI:** 10.1186/s12864-018-5330-5

**Published:** 2019-04-18

**Authors:** Rajesh Kumar, Gandharva Nagpal, Vinod Kumar, Salman Sadullah Usmani, Piyush Agrawal, Gajendra P. S. Raghava

**Affiliations:** 1 Center for Computational Biology, Indraprastha Institute of Information Technology, New Delhi, 110020 India; 2 Bioinformatics Centre, CSIR-Institute of Microbial Technology, Chandigarh, 160036 India

**Keywords:** Chromosomal fragile site, Genomic instability, Database, Chemical inducers, Carcinogenesis, miRNA, DNA elements, Replication stress

## Abstract

**Background:**

Fragile sites are the chromosomal regions that are susceptible to breakage, and their frequency varies among the human population. Based on the frequency of fragile site induction, they are categorized as common and rare fragile sites. Common fragile sites are sensitive to replication stress and often rearranged in cancer. Rare fragile sites are the archetypal trinucleotide repeats. Fragile sites are known to be involved in chromosomal rearrangements in tumors. Human miRNA genes are also present at fragile sites. A better understanding of genes and miRNAs lying in the fragile site regions and their association with disease progression is required.

**Result:**

HumCFS is a manually curated database of human chromosomal fragile sites. HumCFS provides useful information on fragile sites such as coordinates on the chromosome, cytoband, their chemical inducers and frequency of fragile site (rare or common), genes and miRNAs lying in fragile sites. Protein coding genes in the fragile sites were identified by mapping the coordinates of fragile sites with human genome Ensembl (GRCh38/hg38). Genes present in fragile sites were further mapped to DisGenNET database, to understand their possible link with human diseases. Human miRNAs from miRBase was also mapped on fragile site coordinates. In brief, HumCFS provides useful information about 125 human chromosomal fragile sites and their association with 4921 human protein-coding genes and 917 human miRNA’s.

**Conclusion:**

User-friendly web-interface of HumCFS and hyper-linking with other resources will help researchers to search for genes, miRNAs efficiently and to intersect the relationship among them. For easy data retrieval and analysis, we have integrated standard web-based tools, such as JBrowse, BLAST etc. Also, the user can download the data in various file formats such as text files, gff3 files and Bed-format files which can be used on UCSC browser.

**Database URL:** http://webs.iiitd.edu.in/raghava/humcfs/

**Electronic supplementary material:**

The online version of this article (10.1186/s12864-018-5330-5) contains supplementary material, which is available to authorized users.

## Background

Genomic instability is the hallmark of cancer [1] and several other pathologies such as mental retardation [2]; preferentially occurs at specific genomic regions, known as the chromosome fragile sites (CFSs). CFSs are the specific chromosomal regions that exhibit an increased frequency of gaps and breaks when cells are exposed to DNA synthesis inhibitors [3]. These are specific loci which conserved among human and mouse genomes [4]. CFSs can be classified as rare and common depending on their frequency of induction within the population. Rare fragile sites are induced explicitly by BrdU (Bromodeoxyuridine), and folic acid, thus leading to categorization as BrdU (Bromodeoxyuridine) sensitive and folate-sensitive. In case of common fragile sites, Aphidicolin, 5-Azacytidine, and Distamycin-A are most common inducers [5]. Induced fragile sites are involved in sister chromatid exchange, deletion and translocation [6].

Over the past few years, fragile sites have been realized to be an important aspect of cancer biology, as most of the cancer-related genes occur in the CFSs [7]. The genetic instability at fragile sites often results in aberrant expression of oncogenes and tumor-suppressing genes, a step towards initiation of cancer progression [8]. It has been shown by in vitro studies that translocation, deletion, intra-chromosomal gene arrangement and sister chromatid exchange of cancer-specific genomic regions occur as a consequence of cell treatment with fragile site inducers [9, 10]. Common fragile sites even co-localize with breakpoints and deletions specific to various tumors [11, 12]. Epigenetic alterations such as histone hypo-acetylation and methylation contribute towards genomic instability at CFS [13]. For example, Tumor suppressor gene WWOX located within the FRA16D fragile site is often aberrantly methylated and can be correlated with the development of various tumors such as ovary, prostate, and breast cancer [14, 15]. Micro-RNAs (miRNAs), which are essential for cell survival, cell differentiation, metabolism and cell death, also lies in fragile sites, e.g., FRA4D contains miR-218-1 and FRA5G contains miR-218-2 [16]. The deregulated expression of miRNAs due to chromosomal rearrangement has been associated with cancer-specific events and tumor development [17]. For instance, the differential expression of miR218 due to chromosomal rearrangement is allied with bladder cancer development [18, 19].

In the past, numerous resources have been developed to maintain a wide range of information related to instabilities in genome and chromosomes such as i) TICdb, which contain information about chromosomal translocation breakpoint in human tumors [20], ii) HYBRIDdb, maintains information of hybrid genes in humans [21], iii) dbCRID, contains information about chromosomal rearrangement in diseases [22], iv) chimerDB3.0 is a resource of fusion genes [23] and v) COSMIC is a Catalogue of Somatic Mutations in Cancer [24]. Since the discovery of CFSs, several lines of evidence suggest their involvement in human disease progression, as CFSs are preferred sites for exogenous DNA insertion, chromosomal translocation, re-arrangement, and breakpoint. But there remains a significant gap in the current understanding of the human CFSs and the functional components lying within these sites such as miRNAs. One possible reason could be a lack of attempts to associate and integrate divergent CFS studies in literature, leading to the absence of a complete view for the study of these versatile human genomic regions. Best of our knowledge there is no database in the literature that maintains information on the human CFSs, even though fragile sites are core genomic regions responsible for instability and diseases. Considering the importance of CFSs and to complement other related existing resources we have developed a database related to human CFSs.

## Construction and content

### Data collection and compilation

The relevant articles were collected from PubMed by searching the combination of strings “fragile sites”, “chromosomal fragile region”, “chromosomal breakage region”, “genomic breakpoints and fragile sites”, “genomic breakpoints”, to collect comprehensive information about fragile sites. The total number of hits found using the above-searched terms were 4068. Each abstract was manually examined for the information regarding human CFSs, which includes fragile site name, coordinate, cytoband, inducer, frequency, type and technique used for their identification. Final data was gathered from 83 PubMed articles. Some of the fragile sites are not characterized at the molecular level, for them, regions corresponding to cytoband positions were considered as fragile sites. Coordinate information is considered to define the position for molecularly cloned fragile sites as per literature.

### Data curation and organization

**Fig. 1:**
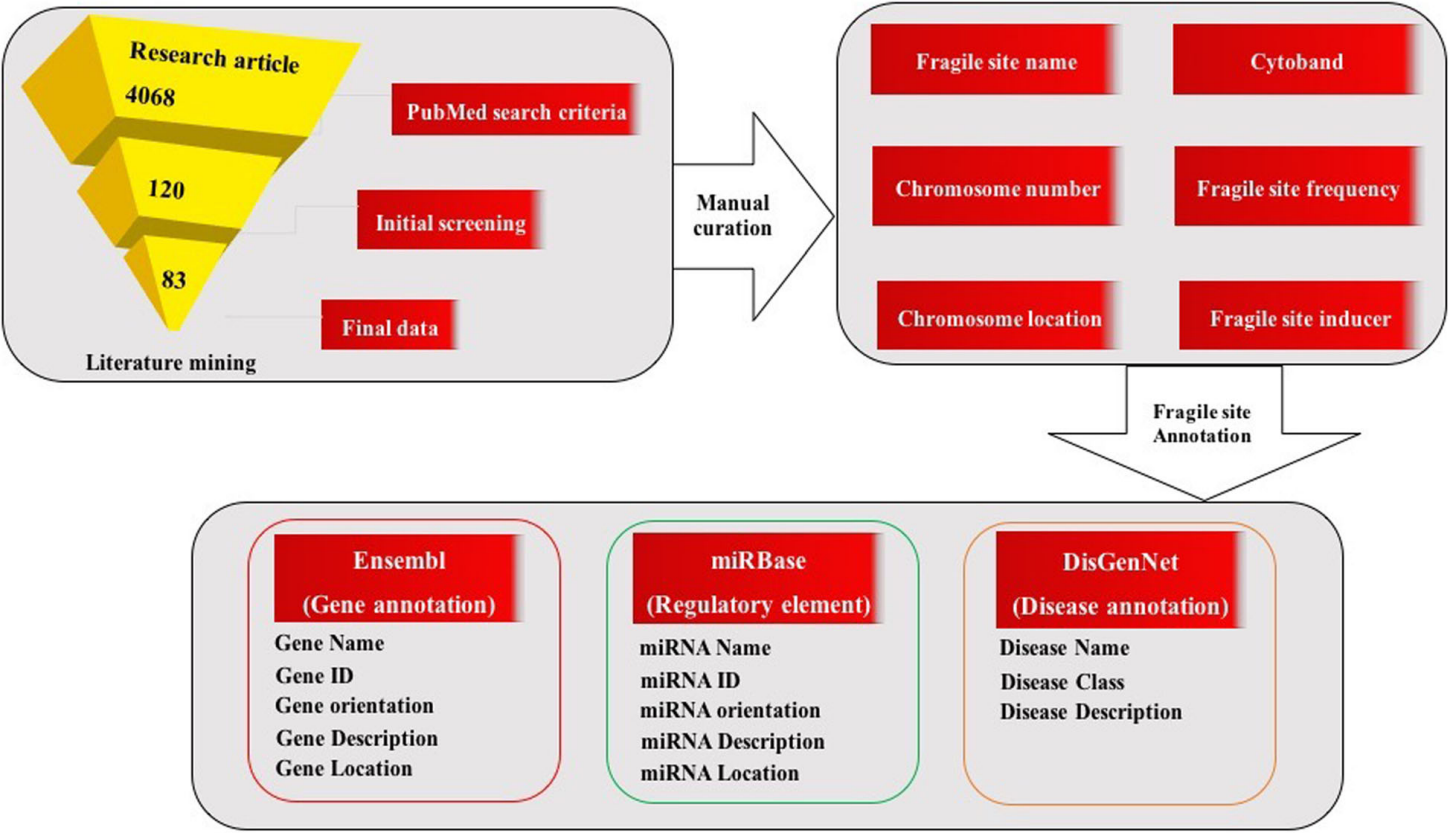
A Schematic representation of HumCFS database architecture

Primary information about the human CFSs, such as the name of the fragile site, chromosomal location, cytoband, type, frequency, technique used for their characterization and identification, and the chromosome number were manually extracted from the PubMed articles. Apart from the primary information, we have processed three levels of annotation for each fragile site, which provides a complete and well-annotated picture of the fragile sites. First level annotation includes mapping of protein-coding genes on the chromosomal coordinates of fragile sites. For this, data from human reference genome assembly (GRCh38/hg38) (http://hgdownload.soe.ucsc.edu/goldenPath/hg38/chromosomes/) was downloaded from Ensembl. An in-house Python script was used to extract the data from human genome reference assembly and to map on the coordinates of CFSs. Second level annotation of fragile sites includes information about miRNAs lying in fragile sites. Human miRNAs data from miRBase [25] was downloaded and mapped on the fragile site by using a Python script. The third level of annotation provides information about human CFSs gene and their association with diseases. Each gene present in the database was linked to the disease category by taking data from DisGenNET [26] database. Finally, information encompassing 4921 protein-coding genes, 917 miRNA and association of genes to human diseases, was systematically and comprehensively compiled in HumCFS. The complete architecture of HumCFS database is compiled in Fig. 1.

### Web server development

Web interfaces work on client-side standard web browsers and provide easy access to HumCFS by various search functions. HumCFS database has been built using a standard platform based on the Linux-Apache-MySQL-PHP (LAMP). Red Hat Linux (version 6.2) as the operating system, MySQL (version 14.12) for managing the data and Apache (version 2.2.17) as the HTTP server, were used for hosting this database. HTML5, PHP5, CSS3 and JAVA scripts have been used for developing the mobile and tablet compatible web server.

## Utility and discussion

### Search tools

The search tools allow users to query the database by performing a search in any field of the database like fragile site name, cytoband, chromosomal coordinate, type of inducers, miRNA associated with the gene etc. This module also allows a user to customize the output according to the field selected for display.

### Browse tools

**Fig. 2:**
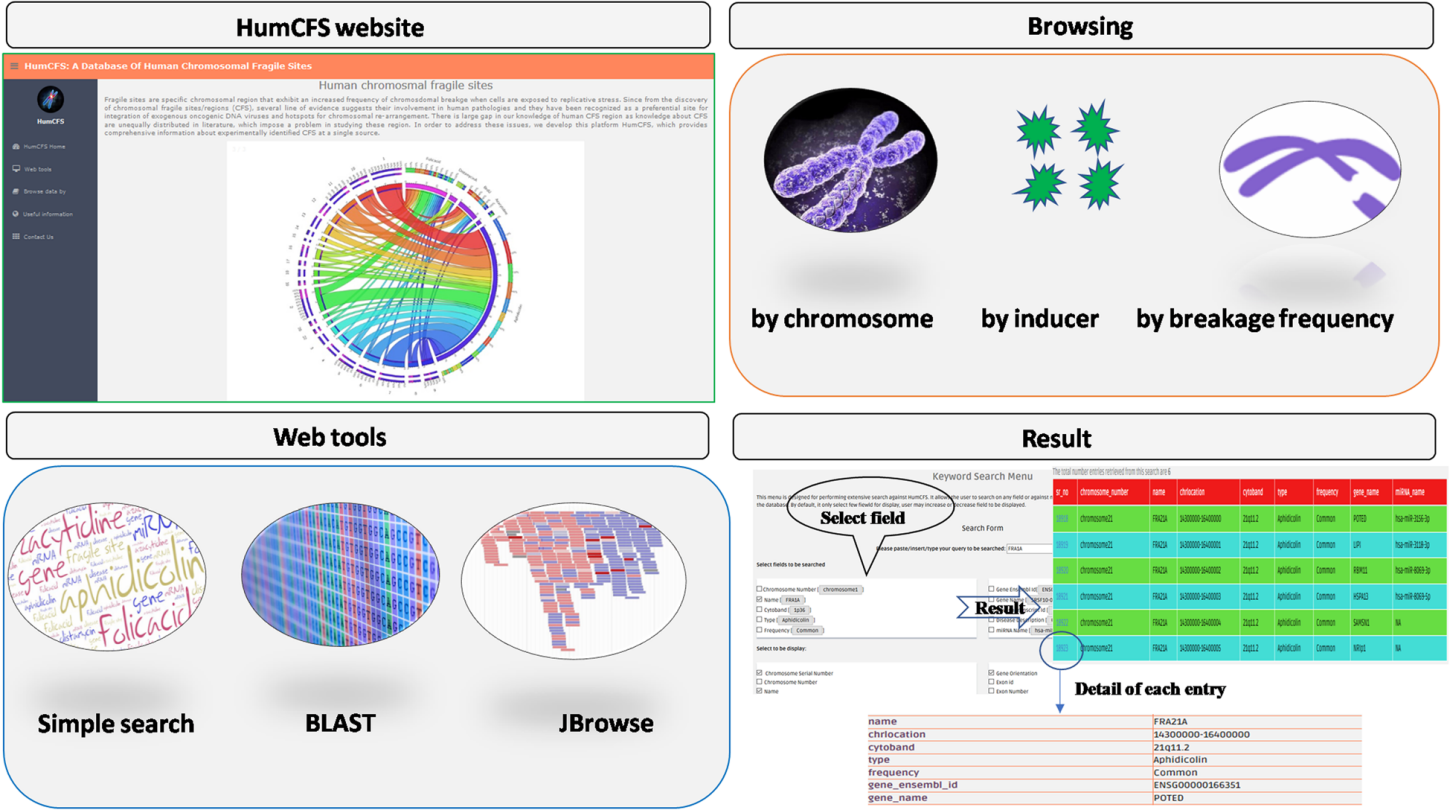
A schematic representation of tools implemented in HumCFS database

HumCFS is equipped with browsing facility that allows accessing data on major fields; as (1) chromosome number (2) fragile site inducer (3) frequency of breakage (4) Moonlight disease search (Fig. 2). Browsing by chromosome number provides information about the number of fragile sites, genes and miRNAs lying in the fragile sites in each chromosome. A user can also retrieve all the genes and miRNAs lying in a specific fragile site. Browsing by inducer allows a user to look for all the human CFSs induced by a particular chemical. Breakage frequency browsing provides a list of all common and rare fragile sites. Many genes present in HumCFS are associated with more than one kind of human diseases. Therefore, to aid in search of this kind of genes, we have provided a ‘Moonlight disease’ module in our database. By utilizing this module, the user may search for a gene, which in addition to link with cancer is also linked with cardiovascular and metabolic disease etc. So this search criterion allows the user to perform a search for genes involved in multiple diseases.

### Website interactive functionality

To assist the user in looking for multiple aspects of the genes and miRNAs present in the database, genes and miRNAs are further linked to other related websites. This includes linking with Ensembl [27], HGNC [28], miRBase [25], Genecards [29], GEPIA [30], COSMIC [24], OMIM [31], EBI-Expression Atlas [32], Human Protein Atlas [33] etc. User can avail this functionality by clicking on Humcfs id. Moreover, the user can also download the data in text, gff3 and bed format. These files can be easily uploaded to the UCSC genome browser as a custom track for other information.

### Sequence alignment

To perform sequence similarity-based search, BLASTN [34] is integrated into the HumCFS database. The user can submit their nucleotide sequence in FASTA format up to 10–1000 lengths. The server performs BLASTN search for the user’s query sequence against the nucleotide sequences of all the fragile sites present in the database.

### Genome browser

**Fig. 3:**
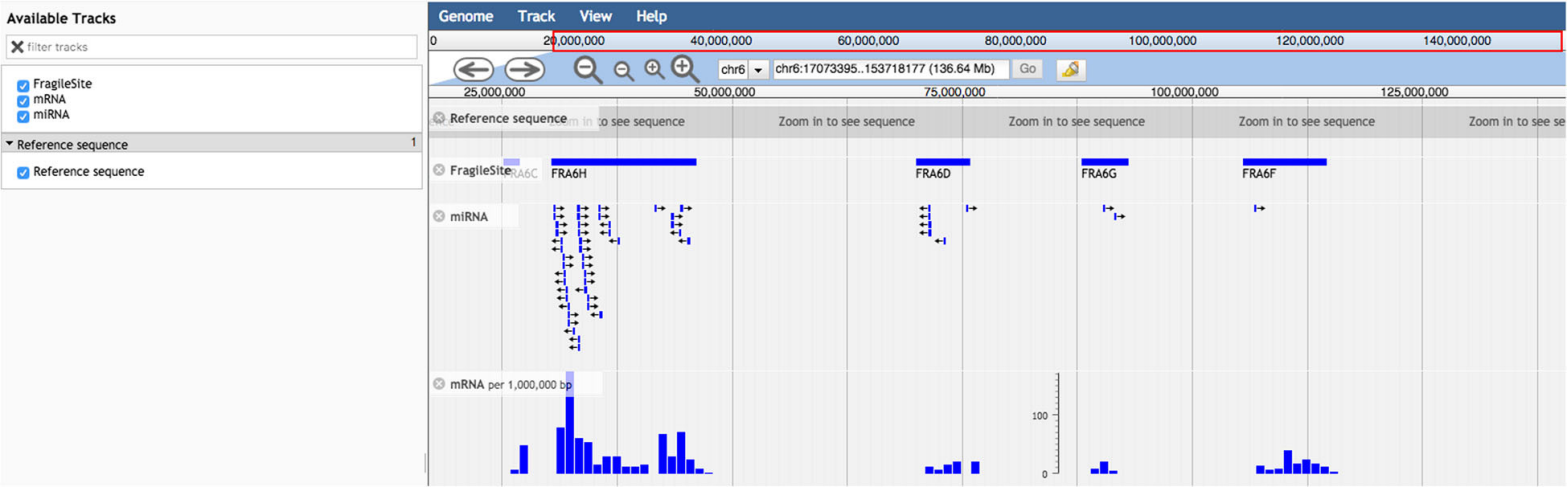
Figure describing data visualization using JBrowse genome browser

We also integrated an interactive ‘Genome browser’ which is powered by JBrowse [35] a JavaScript and HTML5.0 based browser to develop descriptive section using JSON (JavaScript Object Notation data format) which allows fast, smooth, scrolling of fragile site genomic data with unparalleled speed. By clicking on the gene name or the miRNA name, all the information regarding that particular entity including sequence; location, ID, sequence, etc. can be retrieved from the JBrowse (Fig. 3).

## Results

### HumCFS: database statistics, significant findings and analysis

**Fig. 4:**
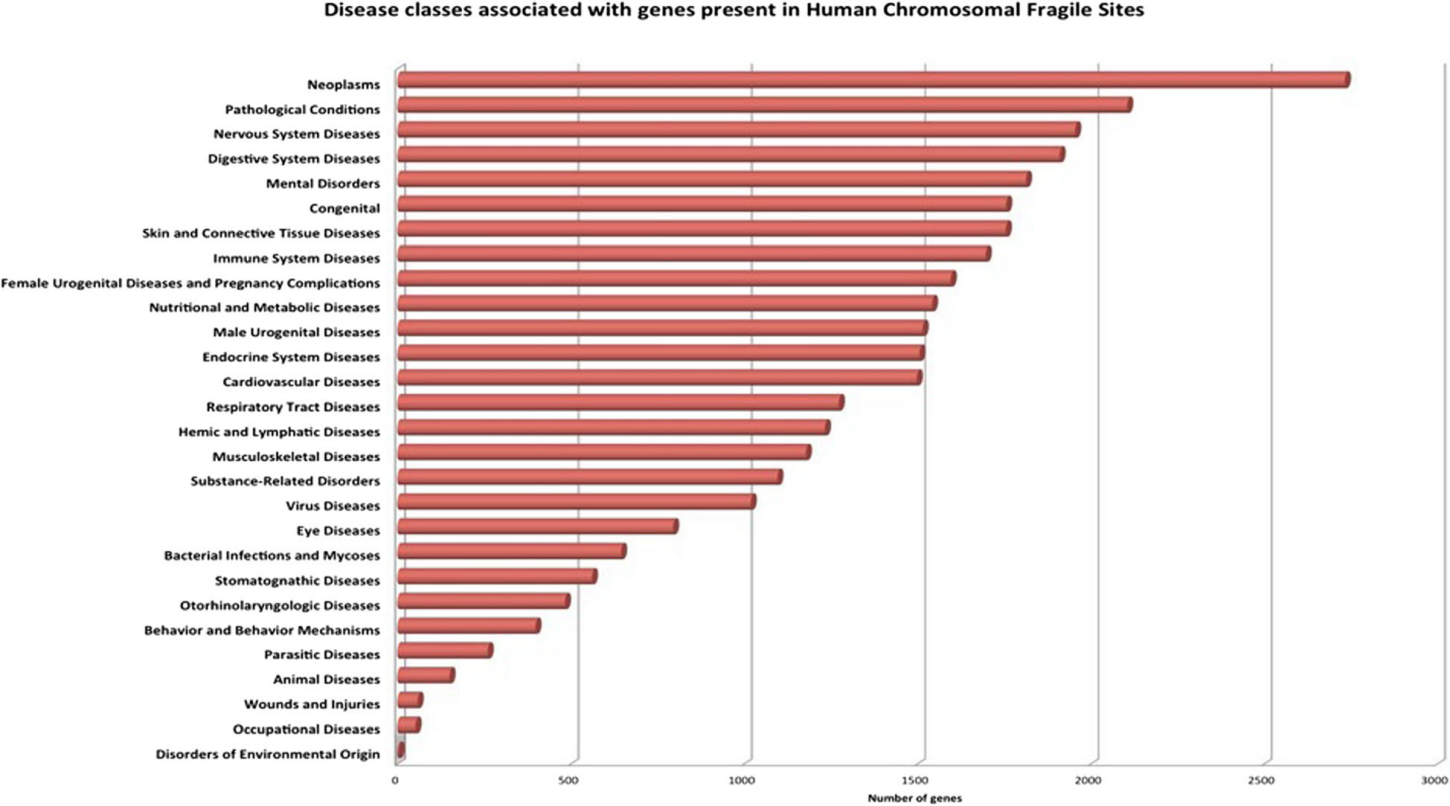
Distribution of genes among various diseases classes recognized by Disease Ontology

**Fig. 5:**
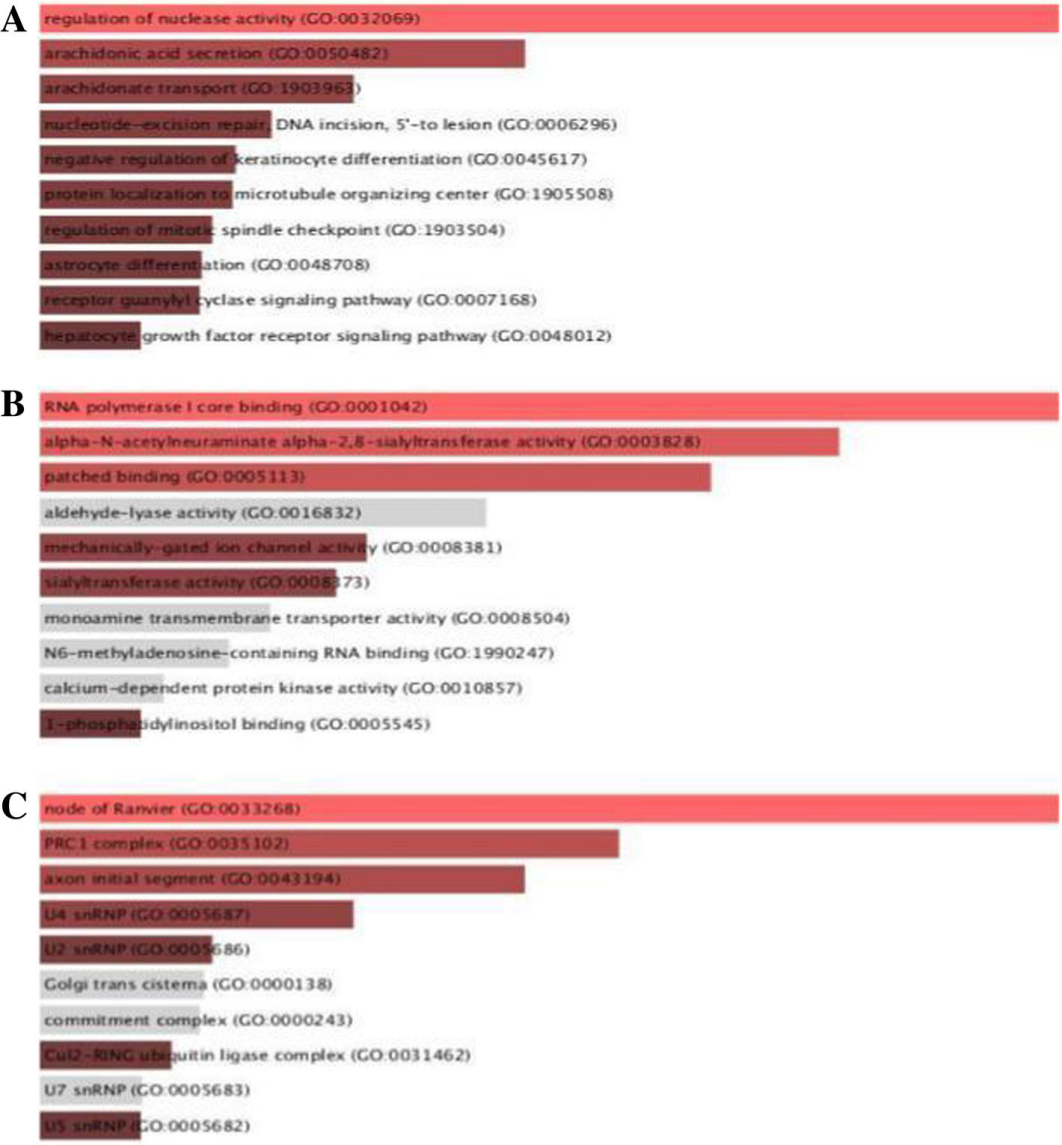
Gene set Enrichment analysis of fragile site genes. **a** Biological Process. **b** Molecular Function. **c** Cellular Components

HumCFS is a unique repository of human CFSs and their genes associated with diseases and human miRNAs. HumCFS comprises of 125 fragile sites lying in entire somatic chromosomes as well as sex chromosome X. These fragile sites contain 4921 protein-coding genes. This analysis reveals that 34.51% of human protein-coding genes lie within the fragile sites which indicate the importance of stability of fragile site is essential for normal gene expression. For example, the FHIT gene present in the FRA3B fragile site is involved in purine metabolism. Aberrant transcript of FHIT gene may lead to carcinoma [36]. Mapping of human miRNAs from miRBase on CFS coordinates, reveals the presence of 917 miRNAs within fragile site. This indicates that approximately 35.04% of human mature miRNA genes coincide with the fragile sites. This reaffirms an important observation that overall distribution of genes and miRNAs within the fragile sites is much higher. The numbers of fragile sites, genes, and miRNAs corresponding to each chromosome are shown in (Table 1; Additional file 1). The analysis reveals that chromosome 19 has the highest number of genes and miRNA in fragile sites, while chromosome 21 has the least number. DisGenNET is one of the largest publicly available collections of genes and variants associated with human disease [26]. Out of 4918 protein-coding genes present within human CFSs, we were able to map 3669 (74.6%) genes to DisGenNET database using HGNC symbols, indicating their association with human diseases. Debacker et al. also reviewed fragile sites in many diseases [37]. Disease ontology analysis of genes presents in fragile sites was done by harboring Disease Ontology database (DO) [38]. A higher number of genes were found to be associated with neoplasm, nervous system disease, pathological conditions, and mental ailments. The distribution of these genes among various disease classes recognised by DO is shown in (Fig. 4). Gene ontology analysis for genes was also done for describing the function of gene products taking part in a biological system by Enrichr [39] (Fig. 5). A higher number of genes was found to regulate nuclease activity. Regulation of nuclease activity is essential to maintain genomic stability since nuclease activity can produce free ends of DNA, can induce DNA recombination which leads to genomic rearrangements [40, 41].

**Table 1:** Distribution of Fragile sites, genes and miRNA among each chromosome

Chromosome No.	Fragile Site	Genes	miRNA
Chromosome 1	13	664	178
Chromosome 2	13	513	78
Chromosome 3	4	110	14
Chromosome 4	5	126	14
Chromosome 5	8	219	36
Chromosome 6	8	293	55
Chromosome 7	11	369	51
Chromosome 8	5	140	38
Chromosome 9	7	68	11
Chromosome 10	6	318	56
Chromosome 11	9	356	70
Chromosome 12	5	321	44
Chromosome 13	5	36	7
Chromosome 14	2	67	9
Chromosome 15	1	55	10
Chromosome 16	5	123	25
Chromosome 17	2	28	5
Chromosome 18	3	42	9
Chromosome 19	2	1091	193
Chromosome 20	2	21	4
Chromosome 21	1	6	4
Chromosome 22	2	41	12
chromosome X	5	3	27

## Conclusion

Genomic instability in the form of chromosomal rearrangements and mutations is characteristic of almost all types of cancer [42, 43]. Two types of factor play important roles in genome instability- one that acts in trans and suppresses genome instability, which includes damage repair mechanism and cell cycle checkpoint inhibitors [44], and the other factor being the chromosomal hotspots for the genomic instability known as fragile sites which are AT-rich sequences, evolutionarily conserved in human and mouse genome and are highly transcribed [4, 45]. But the knowledge about human CFSs is scattered in literature, posing challenges in studying these important regions of the human genome. Considering the necessity of a unified platform, an attempt has been made in the present study to develop a knowledgebase for exploring the human CFSs. In HumCFS, all relevant information regarding CFSs has been compiled in a systematic manner, which will help the researchers to look into a variety of aspects of these important regions of the genome. In the present study information regarding CFSs has been manually curated from research articles and annotated using Ensembl gene files, miRBase and DisGenNET database. We observed chromosome 19 have the highest number of protein-coding genes and miRNAs lying in fragile sites, which is consistent with the previous study [16], and chromosome 13, 14 and 17 show the lowest number of miRNA and protein-coding genes lying in fragile site. This indicates that the distribution of functional elements in the genome is not even, it depends upon the chromosome. Briefly, the user can take benefit from HumCFS in following ways (i) the user can browse a fragile site annotation by one click; this will save time (ii) extract the information about various diseases (iii) visualize genomic regions by genome browser. We believe that HumCFS is a useful resource that will expedite the human CFSs based research.

The current study is an initiative that could pave the way for possible health challenges that could be incurred by the individual due to the chromosomal breakage events. In-depth understanding of the relationship of fragile sites with diseases is a prerequisite for the determination of therapeutic strategies based on the genomic profile of an individual. Thus, in future, detection of chromosomal breakage along with the genomic site of the breakage event could become a part of the genomic profiling of patients that could help in choosing disease management. Although the present study is a comprehensive resource, anticipated to provide an impetus to the fragile site-disease association research and application; diligent efforts are required to apply this knowledge to the prognosis of diseases. This would be possible with the rigorous disease-specific investigation of associated chromosomal breakage events. In literature, most of the studies link CFSs to cancer, but efforts are required for investigating the association of CFSs with other diseases also, as genes related to cardiovascular, metabolic, mental, musculoskeletal, respiratory, nervous systems etc. are also found in human CFSs.

## Additional file

Additional file 1: Circos diagram explaining the number of fragile sites, genes, and miRNA in each chromosome. (letter 1–22 denotes chromosome number, cfs denote chromosomal fragile site. (DOCX 335 kb)

## Abbreviations

CFS: Chromosomal Fragile Sites
DO: Disease ontology
HTML: Hypertext markup language
HumCFS: Human chromosomal fragile site
JSON: JavaScript Object Notation data format
Mbp: Million base pair
miRNA: microRNA
MySQL: Structured Query Language
PHP: Hypertext Preprocessor (earlier called personal home page)
