# Supplementary material for: HumCFS: a database of fragile sites in human chromosomes
Source: BMC Genomics. 2019 Apr 18;19(Suppl 9):985. doi: 10.1186/s12864-018-5330-5 (PMC7402404; doi:10.1186/s12864-018-5330-5)
Supplement: Supplementary file 1 — Circos diagram explaining the number of fragile sites, genes, and miRNA in each chromosome. (letter 1–22 denotes chromosome number, cfs denote chromosomal fragile site. (DOCX 335 kb) [file 12864_2018_5330_MOESM1_ESM.docx]

**Additional File 1:** Circos diagram explaining the number of fragile sites, genes, and miRNA in each chromosome. (letter 1-22 denotes chromosome number, cfs denote chromosomal fragile site.

**
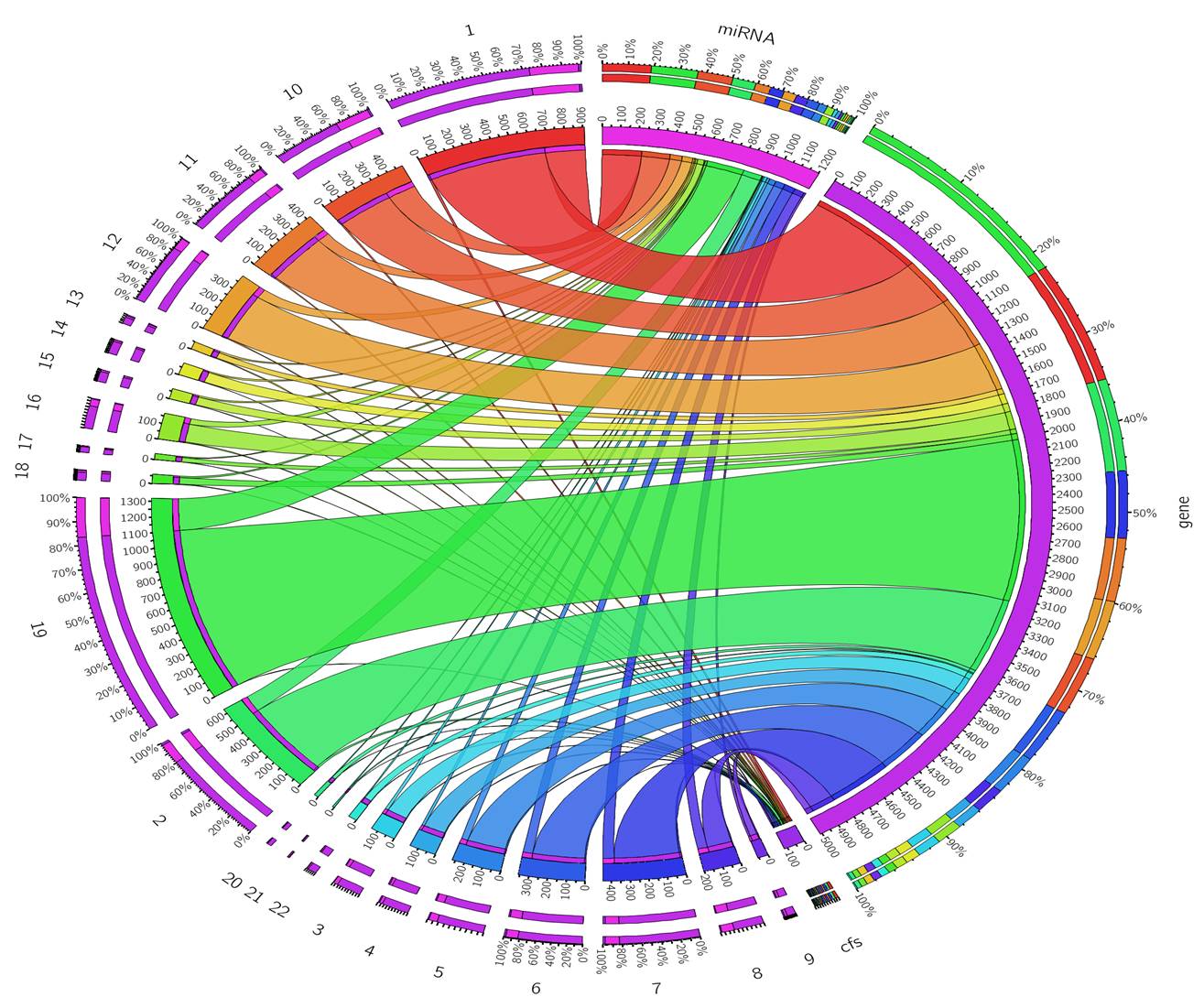
**
